# Not all wavelengths are created equal: disinfection of SARS-CoV-2 using UVC radiation is wavelength-dependent

**DOI:** 10.1099/acmi.0.000276

**Published:** 2021-11-02

**Authors:** Richard M. Mariita, James W. Peterson

**Affiliations:** ^1^​ Crystal IS Inc., an Asahi Kasei company, Green Island, New York, 12183, USA

**Keywords:** Covid-19, Inactivation, SARS-CoV-2, Ultraviolet Disinfection, UVC LED, Wavelength

## Abstract

SARS-CoV-2 is mostly transmitted through close contact with infected people by infected aerosols and fomites. Ultraviolet subtype C (UVC) lamps and light-emitting diodes can be used to disrupt the transmission chain by disinfecting fomites, thus managing the disease outbreak progression. Here, we assess the ultraviolet wavelengths that are most effective in inactivation of SARS-CoV-2 on fomites. Variations in UVC wavelengths impact the dose required for disinfection of SARS-CoV-2 and alter how rapidly and effectively disruption of the virus transmission chain can be achieved. This study reveals that shorter wavelengths (254–268 nm) take a maximum of 6.25 mJ/cm^2^ over 5 s to obtain a target SARS-CoV-2 reduction of 99.9%. Longer wavelengths, like 280 nm, take longer irradiation time and higher dose to inactivate SARS-CoV-2. These observations emphasize that SARS-CoV-2 inactivation is wavelength-dependent.

## Introduction

Towards the end of 2019, a novel viral species from the *Coronaviridae* family, called severe acute respiratory syndrome coronavirus 2 (SARS-CoV-2), which causes Covid-19, began spreading in China [1]. The outbreak ended up becoming a pandemic, leading to a global health crisis [2].

Although SARS-CoV-2 is mostly transmitted through close contact with people by infected aerosols, it can be transmitted via fomites [3]. The SARS-CoV-2 virus can remain viable on plastic, stainless steel, copper and cardboard for 72, 72, 48 and 4 h, respectively [4]. Since ultraviolet subtype C (UVC) light can disrupt nucleic acids (RNA or DNA), UVC light-emitting diodes (LEDs) can be used to disinfect surfaces, thus managing disease outbreak. From the start of Covid-19, some devices equipped with UV have been tested to determine their inactivation efficacy against SARS-CoV-2 [5–9]. Here, we assess the ultraviolet wavelengths that are most effective in inactivation of SARS-CoV-2.

The effectiveness of different wavelengths of UVC irradiance is based upon the dose of energy required to achieve a reduction of an organism, where wavelengths requiring lower dose for the identical reduction are deemed more efficient in their use of radiant energy. The effective wavelength is an important consideration for consumers and design teams because of a relationship between exposure time, performance, lifetime and cost [10], where wavelength has an appreciable impact on inactivation effectiveness, reliability and repeatability of disinfection performances. The effective wavelength for viral and bacterial inactivation varies but is often around 265 nm [11]. This paper compares studies, which sought to determine effectiveness of specific UVC sources, to determine the effective wavelength for SARS-CoV-2 disinfection.

## Methods

The study collected and analysed publicly available results of UVC disinfection obtained through standard methods measuring the inactivation of dry SARS-CoV-2 viral particles. The inclusion-exclusion criteria was to analyse data collected using similar standard methodology: the plaque assay method as it is the most common assay in studies involving SARS-CoV-2 [12]. Some SARS-CoV-2 strains assessed for UVC disinfection include USA/WA1-2020 [5] and SARS-CoV-2/Hu/DP/Kng/19–027, LC528233 [6]. The studies were carried out in either BSL-3 and BSl-4 laboratory environment, with SARS-CoV-2 virus being propagated in appropriate mammalian cells, harvested and standardized prior to use in studies.

The plaque assay method was then used to measure infectious SARS‐CoV‐2 virus particles by quantifying the plaques formed in cell culture during experiments [12]. For example, experiments utilizing 268 nm UVC LEDs at Boston University’s National Emerging Infectious Diseases Laboratory (NEIDL) BSL4, were performed using 100 µl of standardized SARS-CoV-2 (7.33×10^3^ p.f.u. ml^−1^) (USA/WA1-2020) [13], which was plated onto the plastic tissue culture petri dish surface (60 mm) in 5 µl aliquots. The virus was then dried in darkness in the BSC before proceeding. A pair of dishes (one to be irradiated and one control wrapped tightly in aluminium foil) were placed in the chamber of the device and irradiated with UVC for a specific period. Following UVC irradiation, the virus was resuspended in 2 ml high-glucose Dulbecco’s Modified Eagle Medium (DMEM) (Gibco) containing 0.04 mM phenol red, 1×antibiotic–antimycotic (Gibco), 1×non-essential amino acids (Gibco), 1×GlutaMAX I (Gibco), 1 mM sodium pyruvate (Gibco) and 2% foetal bovine serum (FBS) (Gibco). The resuspended virus was then serially diluted from 1×10^0^ to 1×10^–2.5^ using half-logarithmic dilutions before performing a plaque assay as outlined in Honko *et al*. [14]. The plaque assay involved the use of Vero E6 cells maintained in high-glucose DMEM (Gibco) with supplementation before processing as outlined Honko *et al*. [14]. All incubation was done at 37 °C and 5% CO_2_ for a specified period. Finally, staining was done using crystal violet to enable visualization of plaques for enumeration and virus titre determination [14].

## Results and discussion

**Fig. 1.**
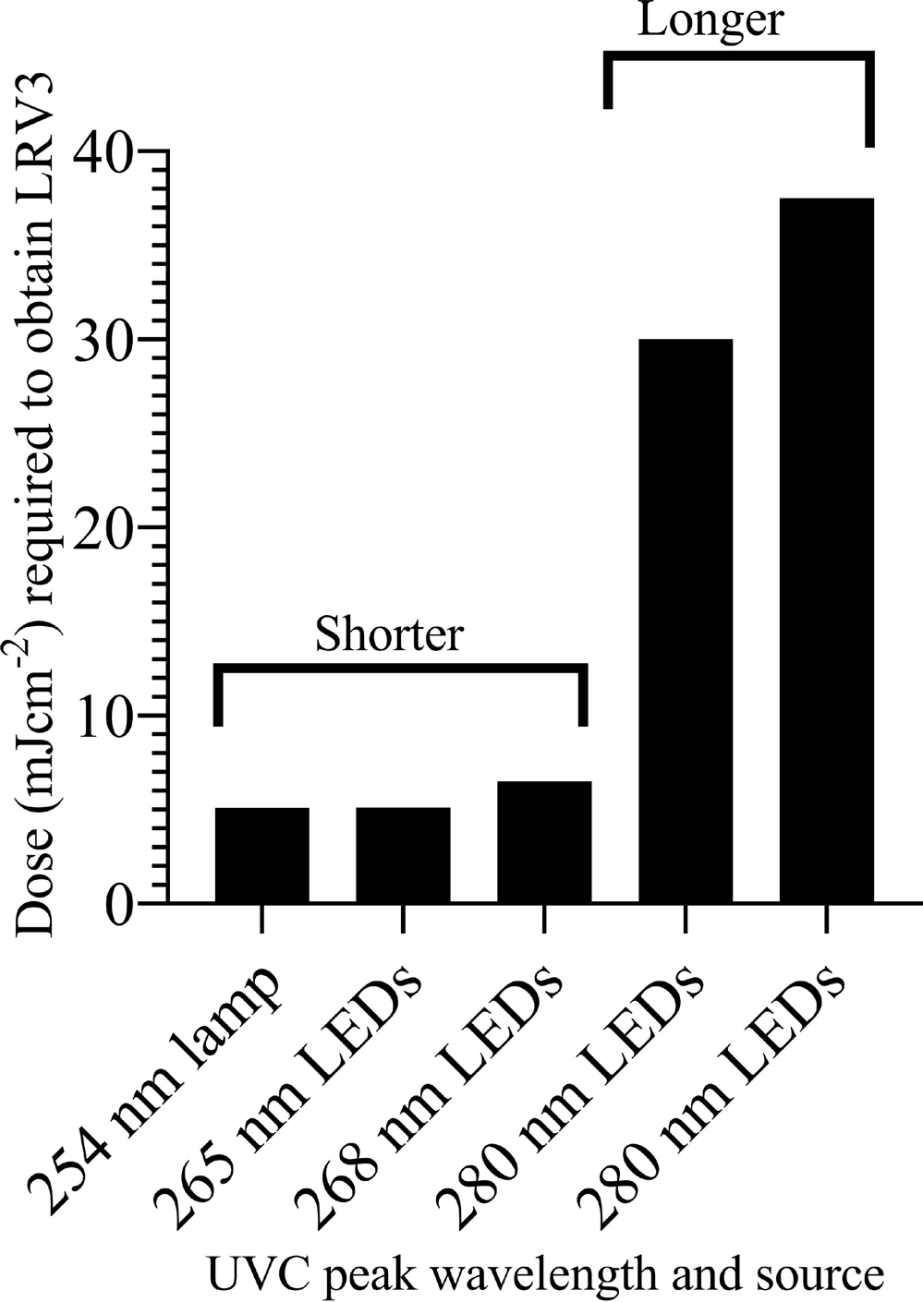
UVC dose required to achieve LRV3 (99.9% reduction of SARS-CoV-2 virus) against peak emission wavelengths from different sources.

Five studies were found that met the criteria. The studies agree with each other in that shorter wavelengths (254–268 nm) require less UVC dose and time to obtain target logarithmic reduction value (LRV). For instance, LRV3 (99.9% reduction of SARS-CoV-2) required 5 mJ/cm^2^ in 5 s at 268 nm (Table 1). At 280 nm, there is a 6.5-fold increase in the required UVC dose to obtain the desired disinfection of LRV3 (Fig. 1). Studies using shorter wavelengths such as 254 nm [5] and 268 nm [7] revealed that it takes less than 10 s of exposure to completely inactivate SARS-CoV-2 virus.

**Table 1.** UVC dose and irradiation time required to obtain given LRV results from different studies using the plaque assay

LED type and wavelength	Host cell	Surface	Dose required to obtain target disinfection performances								
			LRV1		LRV2		LRV3		LRV4		Source
			Time (s)	Dose mJ cm^−2^	Time (s)	Dose mJ cm^−2^	Time (s)	Dose mJ cm^−2^	Time (s)	Dose mJ cm^−2^	
254 nm lamp	Vero E6	Plastic	2	1.7	4	3.4	5	4.3	>6	–	[5]
265 nm LEDs	–	–	–	–	–	–	~5	5.1	–	–	[8]
268 nm LEDs	Vero E6	Plastic	2	3.75	4	5	5	6.25	–	–	[7]
280 nm LEDs	Vero E6	–	5	8.5	10	17	18	31	30	51	[9]
280 nm LEDs	Vero	Plastic	–	–	–	–	10	37.5	–	–	[6]

COVID-19, coronavirus disease 2019; LRV, Log Reduction Value; SARS-CoV-2, coronavirus disease 2019; UVC, UV Subtype C.

The report using coronavirus HCoV-OC43 [15] found only a twofold increase in dose required for 3 log reduction between 267 and 286 nm while our analysis indicates 6.5-fold increase between shorter wavelengths and longer wavelengths for SARS-CoV-2 (Fig. 1). Reports have confirmed the use of UVC LEDs generating around 250–300 nm wavelength to effectively inactivate micro-organisms, including bacteria and surrogates [11], viruses [16] and fungi [17]. Although studies agree that shorter wavelengths are more effective at disinfecting coronaviruses [18], there is a dearth of knowledge specifically on effects of wavelengths on SARS-CoV-2 disinfection as relates to dose and irradiation time. Ideally, a test using a consistent set of LED sources at varying wavelengths on actual SARS-CoV-2 should be performed.

## Conclusion

The study reveals that changes in UVC wavelengths used for the disinfection of SARS-CoV-2 impacts the dose that is required for inactivation and thus irradiation time. The shorter wavelength (254–268 nm) emitting UVC sources are most effective in providing inactivation as they offer more rapid disinfection by requiring a lower total dose than longer wavelength UVC sources. This information is important for consumers and those designing UVC-based disinfection solutions as they may need to consider wavelengths for effectiveness, in addition to tolerance for a given wavelength.
